# Treatment of Refractory Congenital Pseudoarthrosis of Tibia with Contralateral Vascularized Fibular Bone Graft and Anatomic Distal Tibial Locking Plate: A Case Series and Literature Review

**DOI:** 10.3390/children10030503

**Published:** 2023-03-03

**Authors:** Te-Feng Arthur Chou, Ting-Yu Liu, Matthew N. Wang, Chen-Yuan Yang

**Affiliations:** 1 Department of Orthopaedics, Kuang Tien General Hospital, Taichung 433401, Taiwan; 2 Department of Orthopaedics and Traumatology, Taipei Veterans General Hospital, Taipei 112201, Taiwan; 3 Department of Orthopaedic Surgery, Montefiore Medical Center, Albert Einstein College of Medicine, The Bronx, NY 10461, USA; 4 Department of Nursing, Hungkuang University, Taichung 433304, Taiwan

**Keywords:** congenital pseudoarthrosis of tibia, autologous vascularized bone graft, free-fibular bone graft, tibial nonunion, neurofibromatosis, anatomic distal tibia locking plate

## Abstract

Background: Congenital pseudoarthrosis of the tibia (CPT) remains a challenge for physicians. Several treatment options have been proposed, but the standard of care remains inconclusive. In this study, we present three patients for whom the failure of prior treatments was managed with a contralateral vascularized fibular bone graft (VFG) and an anatomic distal tibial locking plate. Methods: Between 2017 and 2021, three patients were referred for failed treatment of CPT. All patients had undergone multiple prior surgeries, including tumor excision and fixation with ring external fixators, plates, and screws. We performed radical tumor resection and reconstruction of bone defects with a VFG. The construct was fixed with an anatomic locking plate, and the patients were followed up for a mean of 45.7 months. Results: All three patients were able to obtain graft union at 19.3 weeks. At the final follow-up, all grafts achieved bony hypertrophy without evidence of bone resorption or local tumor recurrence. There was a mean leg length difference of 8.5 cm preoperatively, compared with 6.3 cm postoperatively. The average lower leg angulation was 7.4 degrees and the average ankle range of motion was 58.3 degrees. The mean VAS score was 0 and the mean AOFAS score was 88.3. No significant complications were noted. Conclusions: Implantation of a VFG and an anatomic distal tibia locking plate can be considered an option for treatment-refractory CPT. Patients can expect to achieve bone consolidation, ambulate as tolerated, and have a low complication rate.

## 1. Introduction

Congenital pseudoarthrosis of the tibia (CPT) remains one of the most challenging conditions to manage [1]. The incidence is reported to be between 1:140,000 and 1:250,000 live births, and is frequently associated with neurofibromatosis (NF) [2]. Other conditions, such as fibrous dysplasia and osteofibrous dysplasia, may also be associated with CPT [3]. Although the cause of CPT remains inconclusive, several hypotheses, such as vascular compromise, soft tissue interposition, and a lack of osteoblastic activity have been considered as the initial pathologic process [3]. CPT is characterized by spontaneous fracture of the tibia in the pediatric population, and the probability of achieving unequivocal union, without refracture, with the index procedure is only about 50% [4]. Therefore, the ideal treatment remains inconclusive [3]. The standard procedure generally involves resection of the pseudoarthrosis site, the pathologic osseous tissue, and the surrounding fibrous hamartoma [3]. The remaining bone defect can be reconstructed with a bone graft, a bone transport, and/or a free vascularized fibular bone graft (VFG), and stabilized with either an internal device or external fixation [5,6,7,8]. A VFG has the advantage of restoring the extensive defect with strut bone and healthy periosteum simultaneously, while fixation methods such as screws, smooth intramedullary (IM) rods, or an external fixator are also used to stabilize the CPT lesion [7,8,9,10]. However, concerns with IM rods include difficulty with nail entry and passage due to osseus deformities, the inadequate purchase of the distal tibial fragment, and the risk of nail exchange due to skeletal development [3]. With the advancements in anatomic plate designs and surgical techniques, most studies have confirmed that tibial plating and intramedullary devices yield comparable outcomes for traumatic fractures of the distal tibia [11]. Moreover, biomechanical analysis revealed that plating can be more advantageous than intramedullary nails for a distal tibial fracture, for which it provides a sufficient amount of axial interfragmentary movement [12]. In this study, we present three CPT patients for whom multiple prior surgical treatments that were performed at other institutions failed. All three patients were managed with radical excision of the diseased bone, while reconstruction with a contralateral VFG and fixation with an anatomic distal tibia locking plate were achieved. In addition, a literature review was completed to review the current management options for CPT.

## 2. Materials and Methods

This is a retrospective review of 3 patients who underwent revision open reduction and internal fixation (re-ORIF) with a VFG and a distal tibia locking plate for non-united CPT. The study was designed in accordance with the Declaration of Helsinki, and approved by the ethics committee of our institution. The surgeries were performed by a single fellowship-trained (microsurgery and trauma) orthopaedic surgeon (CYY) at a regional medical center in Taichung, Taiwan. Each patient had a confirmed diagnosis of neurofibromatosis type 1 (NF-1), a nonunion of CPT that was previously managed with multiple surgical interventions, and had a minimum follow-up of more than 30 months. We excluded patients with other causes of tibia pseudoarthrosis, CPT patients who did not have prior surgical treatments, patients with ongoing infections, and patients that had vascular disorders and coagulopathies. The age, gender, affected side, and number and type of prior surgeries were recorded for each patient.

**Figure 1:**
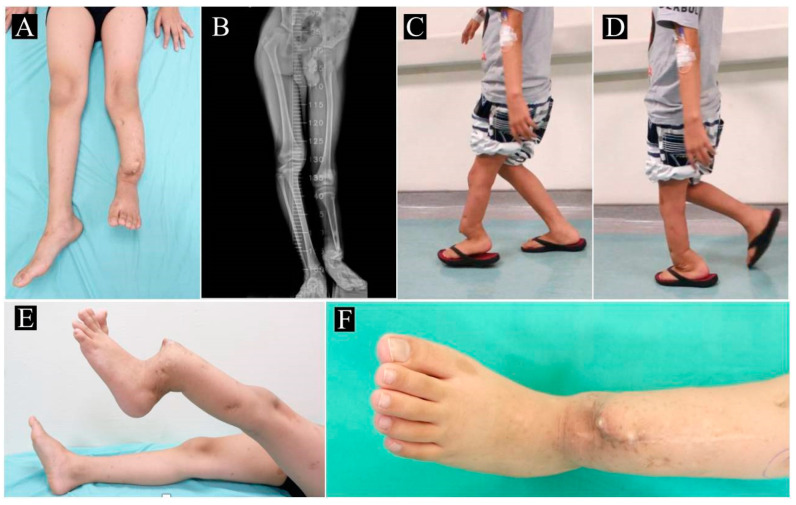
Preoperative evaluation of patient 2. (**A**) Leg length discrepancy (LLD) of 10.2 cm is noted; (**B**) full scanogram confirming the LLD; (**C**) left lower leg deformity prior to weight bearing; (**D**) severe deformity in left lower leg with weight bearing; (**E**) significant angulation upon leg raising; (**F**) near-penetration of the skin due to deformity.

Preoperative evaluation:

All patients underwent standard anteroposterior and lateral radiographs of the lower leg. A full lower-extremity scanogram was also performed to assess for leg length discrepancy (LLD) (Figure 1). The Crawford Classification was used to determine the type of CPT [13]. In addition, a computed tomography angiography (CTA) was performed to evaluate the vascular anatomy (specifically for patent peroneal, anterior tibial, and posterior tibial vessels) of bilateral lower extremities. Ambulating distance was assessed by having the patient ambulate and recording the distance when the patient elected to stop due to discomfort.

**Figure 2:**
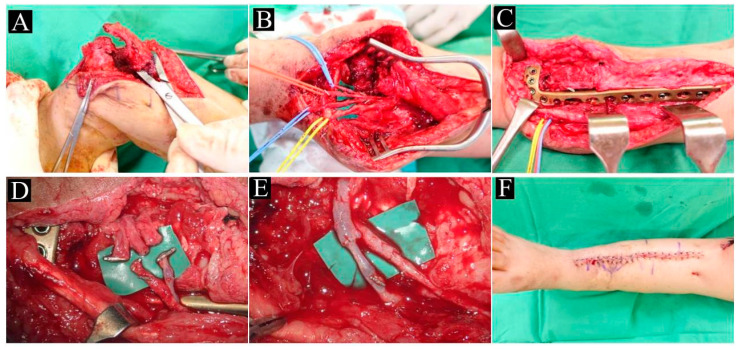
Intraoperative findings: (**A**) the pseudoarthrosis site; (**B**) Radical excision and identification of the anterior tibial artery (red) and veins (blue); (**C**) fibular graft implantation and placement of the anterolateral locking plate; (**D**) preparation for anastomosis of donor and recipient vessels; (**E**) completion of end-to-end anastomosis; (**F**) postoperative wound closure.

Surgical method:

Under general anesthesia, the patient was placed in a supine position and both lower extremities were draped with a sterile technique. A sterile tourniquet was applied over the affected limb and was inflated prior to incision. The pseudoarthrosis site was confirmed and marked under fluoroscopy. A longitudinal anterolateral incision was made just lateral to the tibial crest. The incision extended 7 cm above the fracture site and ended at the level of the ankle joint. The fascia was then excised and blunt dissection was carried down to the CPT. The dense fibrotic hamartoma and diseased periosteum around the CPT lesion (Figure 2A) were radically removed until both ends of the tibia had visible bleeding with healthy bone stock and normal periosteum. The medullary canal was debrided and recanalized to improve graft incorporation. Correction of the antero-lateral deformity was then applied under direct visualization and with the assistance of fluoroscopic images. The size of the defect was measured. The tibial artery and two accompanying veins were identified and marked with vessel loops (Figure 2B). The wound was packed with moist gauze and the tourniquet was deflated.

**Figure 3:**
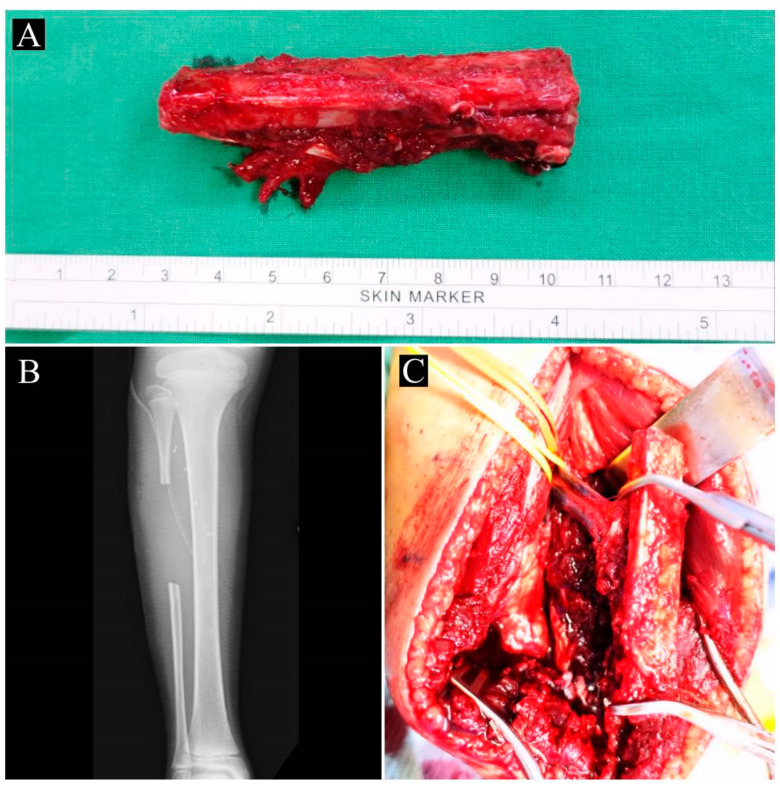
Harvesting the contralateral vascularized fibular graft: (**A**) harvested vascularized graft; (**B**) postoperative X-ray indicating well-preserved fibula beyond the mid-diaphysis region; (**C**) intraoperative image showing the donor peroneal artery and vein and fibular bone graft.

Attention was then turned to the contralateral fibula. A longitudinal incision over the midportion of the lateral calf in line with the fibula was made, and then dissection was performed between the interval of the peroneus muscle and the soleus muscle to expose the fibula. The middle third of the fibula, together with the surrounding healthy periosteum and nutrient vessels (branches of the peroneal artery and two accompanying veins), was harvested. The size of the graft was determined based on the measured defect size (Figure 3A,C). Careful attention was taken to ensure that the graft did not extend beyond the mid 1/3 of the fibula (Figure 3B) in order to preserve ankle joint stability.

The tourniquet was inflated once again. Prior to inserting the VFG into the defect, the proximal end of the VFG was slightly tapered to fit the narrow intramedullary canal of the tibia shaft. The distal end of the tibia was mainly composed of the wider tibial metaphysis in which the graft could slide with ease. A 3.5 mm medial or anterolateral anatomic distal tibia locking plate^®^ (DePuy Synthes, Raynham, MA, USA) was used to stabilize the distal tibia and VFG (Figure 2C). Tension-free end-to-end anastomoses were completed between the donor peroneal artery and the recipient anterior tibial artery, and between the donor peroneal vein and the recipient anterior tibial vein (Figure 2E). The tourniquet was deflated and a patent circulation between the donor and recipient sites was confirmed. A hemovac^®^ (Zimmer Biomet, Warsaw, IN, USA) was inserted. The affected fibula was also treated with radical excision of the hamartoma and the diseased periosteum, then fixed with an appropriately sized Kirschner wire. The incisions were closed and the patient was placed in a below-knee short leg splint.

Postoperative care:

The patient remained immobilized and non-weight-bearing in the short leg splint for 4 weeks. Clinic follow-ups were arranged at 2 weeks, 4 weeks, 6 weeks, and every 4–6 weeks after the 6th week. Plain radiographs and neurovascular exams were completed at each follow-up visit. At 2 weeks, wound inspection and removal of the sutures were completed. At 4 weeks, the splint was removed and the passive range of motion was initiated. Partial assisted weight bearing was also permitted. The patient was allowed to weight-bear as tolerated when the following 3 criteria were met: (1) radiographic evidence of osseus formation filling in the fracture site or bridging callus surrounding the defect, (2) non-tender fracture site, and (3) pain-free upon full weight bearing. At 2.5 years after the operation, the final LLD, lower leg angulation, visual analogue pain scale (VAS), ambulation distance, range of motion in the affected knee and ankle, and AOFAS score were assessed.

## 3. Results

**Figure 4:**
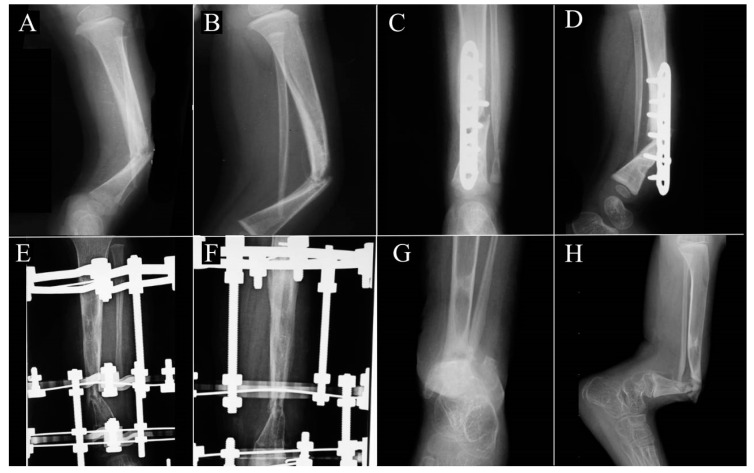
Serial X-rays of patient 2 prior to this correction surgery: (**A**) anteroposterior (AP) and (**B**) lateral views of left lower leg at 2 years old; recurrence of deformity (**C**) AP and (**D**) lateral views after first correction surgery at age 3; the patient was then placed in an Ilizarov frame, as seen in (**E**) AP and (**F**) lateral views, but recurrence occurred at age 4; (**G**) and (**H**) show the patient’s deformity at age 11 upon presenting to our institution.

Between 2017 and2021, a total of three patients with NF-1 complicated with nonunion of CPT were included in this study. All of the patients had a type IV CPT. The mean age was 11 years, the mean follow-up time was 45.7 months, and the mean LLD was 8.5 cm. The baseline characteristics of each patient are shown in Table 1 and the serial preoperative X-rays of patient 2 are shown in Figure 4. 

### 3.1. Intraoperative Findings

The procedure type and intraoperative findings are shown in Table 2. The mean operative time was 20 h. Patient 1 had a CVG graft size of 10 cm, and the tibia was fixed with an anatomic medial distal tibia locking plate ^®^ (DePuy Synthes, Raynham, MA, USA). Patient 2 underwent a similar procedure, aside from using an anterolateral distal tibia locking plate ^®^ (DePuy Synthes, Raynham, MA, USA). In addition, because of significant soft tissue contracture due to multiple prior surgeries and chronic LLD, we performed V-Y Achilles tendon lengthening to accommodate the soft tissue contracture. We were able to gain approximately 5 cm of soft tissue lengthening after the V-Y advancement. Patient 3 required a two-stage procedure due to secondary skin contracture after prior surgeries. The first stage was a CPT excision and a tibia-lengthening procedure with a Taylor spatial frame. Over a 5-week period, the TSP was adjusted daily and we were able to lengthen the tibia by 4 cm. This was deemed within an acceptable range. The patient subsequently underwent a definitive second-stage procedure with VFG implantation and fixation with a similar anterolateral distal tibia locking plate. All of the patients were able to achieve primary skin closure (Figure 2F).

### 3.2. Postoperative Follow-Up

**Figure 5:**
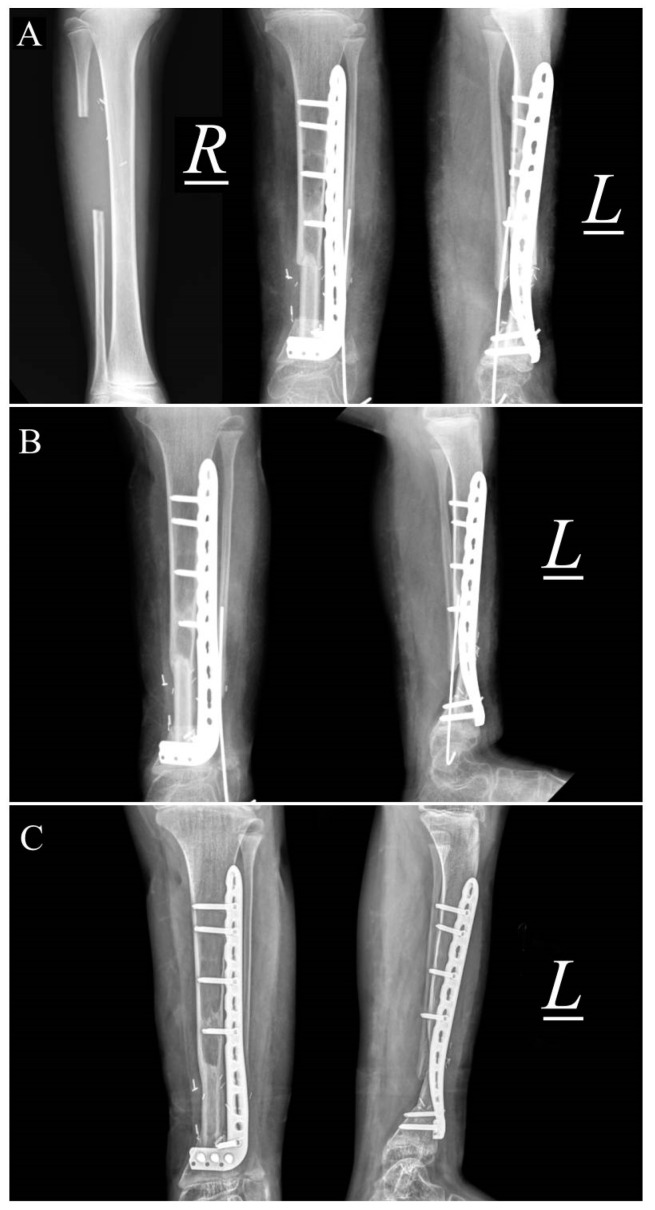
Postoperative follow-up X-ray images of patient 2: (**A**) postoperative 4 weeks with R indicating the right lower leg and L indicating the left. Both anteroposterior (AP) and lateral views are shown for the left. (**B**) AP and lateral X-ray of left lower leg at 3 months. (**C**) AP and lateral X-ray of left lower leg at 12 months.

**Figure 6:**
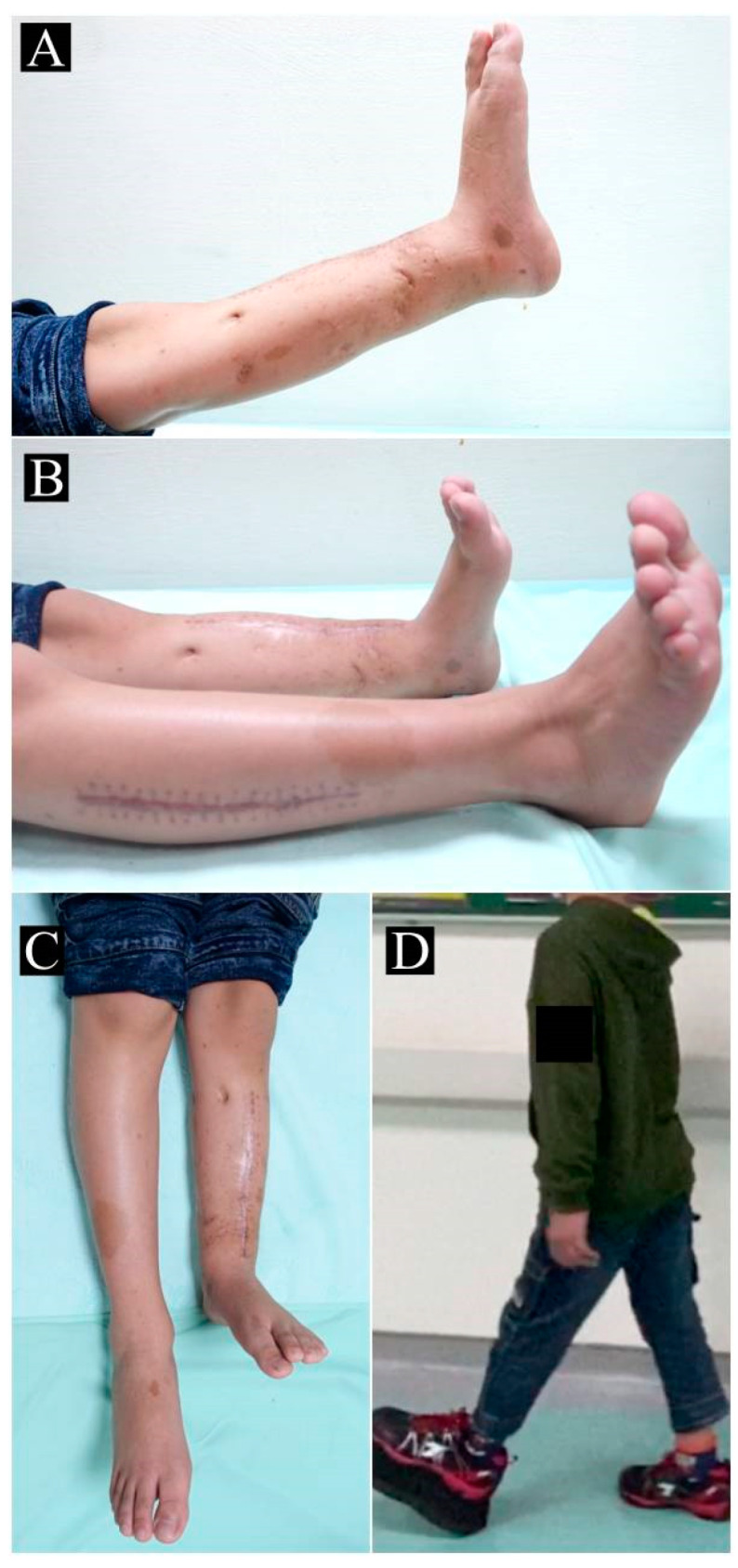
Postoperative clinical images for patient 2 at postoperative 30 months. (**A**) The patient is able to fully extend his knee with no gross angular deformities in the left lower leg. (**B**) Range of motion in bilateral ankles is preserved. (**C**) Leg length discrepancy around 7.5 cm is noted. (**D**) With a 5 cm shoe lift, the patient is able to ambulate with minimal discomfort.

The postoperative information for each patient is shown in Table 3. All three patients were able to achieve bone consolidation at a mean of 19.3 weeks. This was maintained to the final follow-up for all three patients. All of the fibular grafts incorporated well with bony hypertrophy without evidence of bone resorption or local CPT recurrence (Figure 5). There was a mean postoperative leg length discrepancy of 6.3 cm. The average lower leg angulation was 7.4° and the average ankle range of motion was 58.3 degrees. The mean VAS score was 0 and the mean AOFAS score was 88.3. Figure 6 shows clinical images of patient 2 at postoperative 30 months.

**Figure 7:**
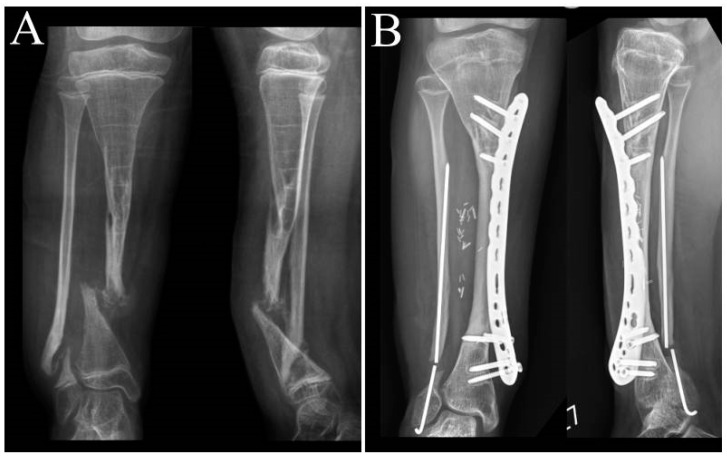
Radiographs of patient 1: (**A**) preoperative AP and lateral radiographs and (**B**) postoperative AP and lateral radiographs at 53 months after the surgery.

**Figure 8:**
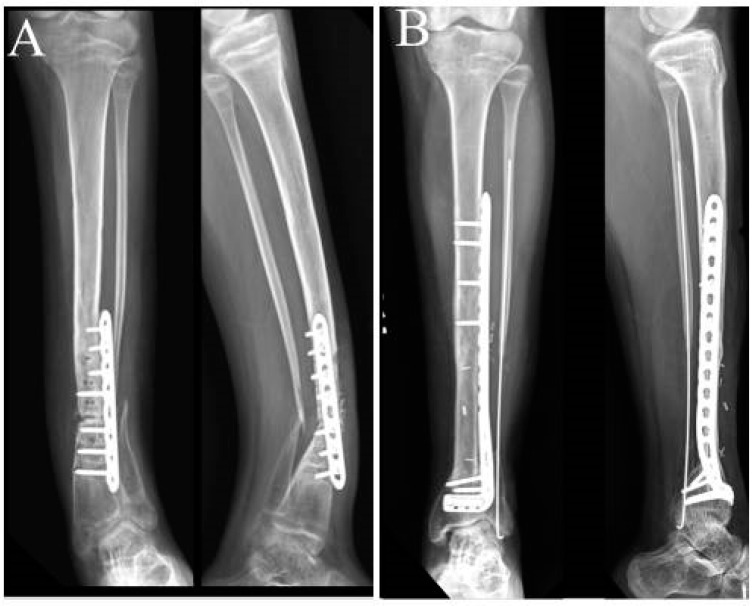
Radiographs of patient 3: (**A**) preoperative AP and lateral radiographs and (**B**) postoperative AP and lateral radiographs at 18 months after the surgery.

Patient 1 had a final LLD of 8.5 cm with a 15° right lower leg valgus deformity. Although the tibia remained consolidated, the fibula was complicated with pseudoarthrosis which was starting at the 20-month follow-up. At the final follow-up (65 months), the patient had a VAS score of 0, and was able to ambulate without restrictions. Her knee ROM was full, and ankle ROM was 75°. For patient 2, bone consolidation was noted at 20 weeks, and his residual LLD was 7.5 cm with 4.7° lower leg angulation. At the final follow-up (42 months), he was able to walk without pain (VAS 0), and was able to walk for 1000 m. However, he continued to have a progressing LLD and currently requires a 5 cm shoe lift. Since his physeal was preserved and he continues to grow taller, he will potentially require corrective surgery in the future. The postoperative clinical images are shown in Figure 6. Patient 3 achieved bone consolidation at 21 weeks. At his final follow-up (30 months), his residual bone defect was 3.0 cm with 2.3° lower leg angulation, and he was able to ambulate without pain or restrictions. The ESF incisions healed without complications. No significant disability-causing complications such as neurovascular deficits, wound dehiscence, limb edema, or donor site morbidity (e.g., peroneal nerve injury, knee or ankle joint instability, or lower extremity weakness) were noted. The preoperative and postoperative radiographs of patients 1 and 3 are displayed in Figure 7 and Figure 8.

## 4. Discussion

In this study, we presented three patients who had NF-1 complicated with repeated failed surgical treatment of CPT, and each subsequently underwent a successful revision surgery with VFG implantation and anatomic distal tibia locking plate fixation. The most significant finding was that all three patients were able to achieve bone consolidation at a mean of 19.3 weeks, and were able to ambulate without pain and restore good ankle function by the final follow-up (mean 45.7 months). In addition, there was no evidence of recurrence during our follow-up period.

There are two main pathologic processes in CPT. The first is pathobiological, in which there is diseased soft tissue (e.g., hamartoma formation and pathologic periosteum) leading to osteolysis and vascular compromise in the local region [4]. In addition, a pathomechanical process occurs, leading to angular deformities and atrophic bone ends [4]. Therefore, treatment options that can address both pathologies is required. In the current literature, the treatment options for reconstruction following the radical excision of CPT lesions are (1) a bone graft and internal fixation with an IM rod or ring external fixator, (2) bone transport, and (3) free VFG transfer with (4) amputation reserved for refractory cases [4]. Although the optimal treatment remains inconclusive, VFG implantation theoretically provides structural support with a healthy periosteum, sufficient length, low donor site morbidities, and reliable circulation that can hasten the healing of the CPT site [3]. In two case series reports, the bone consolidation rate was reported to be near 94–100% with a very low recurrence rate [8,14]. Our results are in agreement, as we were able to achieve consolidation at an average of 19.3 weeks with no evidence of recurrence up to at least 30 months after the surgery. In our series, the short- to mid-term results are promising, but the long-term results remain to be determined.

When treating CPT, one of the most important factors is to apply rigid fixation, in order to maintain axial, rotational, and angular stability at the CPT site [4]. Most studies have favored the use of IM rods, ring external fixators, and screws as the fixation method [6,7,8]. The screws were the weakest mechanically, and ring external fixators have disadvantages such as Schanz screws/wires loosening or pin tract infection after long-term usage. The IM rods seemed to be an option with better fixation stability; however, in the refractory CPT cases, the lesion sites were mostly located in the very distal tibia and fibula [4,14]. This makes it very difficult to establish reliable mechanical stability with only IM devices or ring external fixators in the small distal tibia fragment after extensively resecting the fibrous hamartoma. Therefore, our experience was that fixation with a plate system was more favorable.

In this study, we elected to use distal tibia anatomic locking plates to provide a rigid construct (avoiding rotational and angular deforming forces) in order to reduce refracture and malalignment [15,16]. In the past, controversies with plating caused concern about higher failure rates. However, most of these studies utilized conventional non-locking plates, which have different mechanical properties, and the plates were generally bulkier, which would further increase soft tissue tension and obscure the anastomosing sites [7,17]. Interestingly, the three patients presented in our series were previously treated with straight-type non-anatomic locking plates^®^ (DePuy Synthes, Raynham, MA, USA), which all failed due to distal metaphyseal fragment loosening and screw pullout. With the development of low-profile 3.5 mm anatomic distal tibia locking plates with more metaphyseal screws, these concerns seem less problematic [4]. In our study, anastomosis was achieved between the peroneal vessels on the VFG and the recipient anterior tibial vessels, which are located in the anterior leg compartment. Since the plates were relatively low-profile, they did not obscure the surgical field and we were able to complete the anastomosis with relative ease. Moreover, a lower-profile plate placed less tension on the surrounding soft tissue. This is crucial in patients with CPT to allow for wound closure.

In our first patient, a medial distal tibia LCP^®^ (DePuy Synthes, Raynham, MA, USA) was used. Although consolidation of the CPT was achieved, insufficient mechanical support on the lateral side, together with pseudoarthrosis of the ipsilateral fibula, progressively caused a valgus deformity of up to 15 degrees to develop. In addition to the minimal soft tissue coverage on the medial aspect of the lower leg, especially in these patients with a history of multiple surgeries, this made medial plates less desirable [18]. Therefore, an anterolateral distal tibia LCP^®^ (DePuy Synthes, Raynham, MA, USA) was used in the subsequent patients. In comparison to the medial plate, with a transverse metaphyseal screw axis, the anterolateral plate had metaphyseal screws in the anteroposterior axis, which could potentially resist screw pullout from the valgus deformity. In the diaphyseal region, the laterally placed design of the anterolateral plate provided a stronger buttress force than the medial plate, which is crucial to prevent further valgus deformity and malunion [19]. In the clinical study of distal tibia fracture with comminuted fibular fracture, the anterolateral plates also showed mechanical superiority to the medial plate [19].

In our experience, the limitations of anterolateral plating with a 3.5 mm fixed-angle distal tibia LCP are the plate width, locking screw axis, and its proximity to growth plates in the metaphyseal area. Therefore, some patients may require a slight adjustment of the plate placement, or the potential removal of one or two screw holes to adequately fit each individual patient. Moreover, the locking mechanism increased the fixation stability, but the fixed-angle screw axis and proximity to growth plates may pose a challenge during the placement of the locking plate. To prevent intraoperative physeal injuries, the locking sleeves can be preloaded onto the screw hole to help predict the metaphyseal screw axis while meticulously adjusting the plate positions. These advantages could be improved by adopting a newer version of the distal tibia LCP with more metaphyseal screw holes, a smaller diameter (2.7 mm) locking screw, variable-angle screw axis, and a lower profile design than the currently used 3.5 mm LCP systems [20].

Residual deformities after CPT correction are a frequent problem [4,21]. For instance, tibia diaphyseal malalignment, LLD, ankle arthritis, ankle valgus deformity, and calcaneal deformities can all occur [4,21]. In particular, LLD can be problematic for these patients, with the incidence said to be as high as 56% [21]. The reason for this is growth arrest secondary to the pathologic process of CPT, as well as multiple operations that could lead to physeal injuries [3,21]. In our series, the average LLD at the final follow-up was 6.3 cm. These patients were managed with a shoe lift that could partially compensate for this difference. Current options for management include shoe lifts for defects < 5 cm; epiphysiodesis of the contralateral femur, tibia, or both in defects < 5 cm; and lengthening procedures with ESF or the Ilizarov technique in defects > 5 cm [3,21]. Given the high incidence of LLD, subsequent procedures to address the LLD should be informed to the patients. Moreover, a high rate of pseudoarthrosis can be seen at these lengthening sites [21], although the significance of this remains unknown. Despite having a residual LLD up to 8.5 cm in our series, all three of our patients tolerated their LLD well with good functional outcomes (average AOFAS score 88.3) and did not require further lengthening or corrective procedures.

A potential alternative procedure to consider is the induced membrane technique (IMT) previously applied by Masquelet et al. for the treatment of septic nonunion of the tibia [22]. In a retrospective study performed by Vigouroux et al., the authors followed 18 patients for a mean of 9.5 years and revealed comparable results with patients who underwent VFG implantation [9]. The IMT, theoretically, can reduce donor site morbidity while providing a biological chamber that contains growth factors and angiogenetic factors that can promote osteointegration of the graft [9]. However, this procedure requires two stages, and further long-term results are required to further confirm the efficacy of this method.

This study is not without limitations. Given the small number of cases we presented, inherent concerns related to a small sample size are inevitable. In addition, we did not have a control or comparison group to validate our results against. Nonetheless, our results seem in line with the current literature. Moreover, given the limited resources available at smaller institutions, all of the surgeries were performed by a single surgeon. This led to a longer operative time and can be a burden for both the surgeon and the patient. Lastly, harvesting the VFG and the microvascular anastomosis of the graft can be technically demanding and requires a learning curve. Nonetheless, VFG implantation appears to have a favorable outcome in this subset group of challenging patients.

## 5. Conclusions

Treatment of refractory congenital pseudoarthrosis of the tibia with a contralateral vascularized fibular bone graft and anatomic distal tibial locking plate fixation appears to have satisfactory results at mid-term follow-up.

## Figures and Tables

**Table 1:** Patient demographic.

Patient	Age (Years)	Gender	Laterality	Pseudoarthrosis *	LLD (cm)	Ambulation (Meters)	Prior Surgeries	F/u Time (Months)
1	9	F	R	Type IV	11.1	50	ORIF:2	65
							ROI:2	
							BG:1	
2	11	M	L	Type IV	10.2	10	ORIF:1	42
							IEF:3	
							ROI:2	
3	13	M	L	Type IV	4.1	30	ORIF:4	30
							ROI:1	
							BG:2	
Mean	11				8.5	30	6	45.7

* Classified based on Crawford classification; LLD—limb length discrepancy; F/u—follow-up; ORIF—open reduction and internal fixation; IEF—Ilizarov external fixation; ROI—removal of implant; BG—bone graft.

**Table 2:** Intraoperative findings.

Patient	Procedure	Graft Fixation	Graft Length (cm)	Operative Time
1	CPT excision and reconstruction with contralateral VFG	Anatomic medial distal tibia locking plate	10	16.5 h
2	CPT excision, Achilles tendon lengthening and reconstruction with contralateral VFG	Anatomic anterolateral distal tibia locking plate	7	20.5 h
3	1st stage: CPT excision and tibia lengthening with TSP 2nd stage: reconstruction with contralateral VFG	Anatomic anterolateral distal tibia locking plate	10	23 h

CPT—congenital pseudoarthrosis of the tibia; VFG—vascularized fibular graft; TSP—Taylor spatial frame.

**Table 3:** Postoperative radiologic and functional outcomes (at 30 months).

Patient	Graft Consolidation	Final LLD	Angulation	VAS	Ambulation Distance	ROM	AOFAS Score
1	17 weeks	8.5 cm	valgus 15°	0	Without limitations	Knee: full/Ankle: 75°	88
2	20 weeks	7.5 cm	valgus 4.8°	0	1000 m	Knee: full/Ankle: 50°	82
3	21 weeks	3.0 cm	valgus 2.3°	0	Without limitations	Knee: full/Ankle: 50°	95

LLD—leg length discrepancy; VAS—visual analogue scale; AOFAS—American Orthopedic Foot & Ankle Society.

## Data Availability

Data are unavailable due to privacy or ethical restrictions.
